# Double Connection of Left-Sided Partial Anomalous Pulmonary Vein Return in a Young Man

**DOI:** 10.1016/j.jaccas.2024.102398

**Published:** 2024-06-05

**Authors:** Leila Bigdelu, Ossama Maadarani, Ali Azari, Ali Heidari-Bakavoli, Zouheir Bitar

**Affiliations:** a Division of Cardiovascular Medicine, Vascular Surgery Research Center, Mashhad University of Medical Sciences, Mashhad, Iran; b Critical Care Unit/Internal Medical Department, Ahmadi Hospital–Kuwait Oil Company, Ahmadi, Kuwait; c Department of Cardiovascular Imaging, Razavi Hospital, Mashhad, Iran

**Keywords:** computed tomography, congenital heart defect, echocardiography

## Abstract

Double connection of partial anomalous pulmonary venous return is a very rare congenital anomaly where at least one pulmonary vein, but not all, drains into the left atrium and systemic venous circulation with subsequent left to right shunt.

## Graphical Abstract

**Figure:**
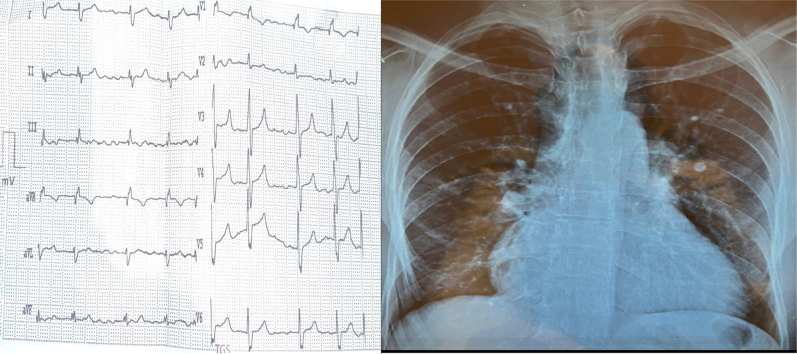

Partial anomalous pulmonary venous return (PAPVR) is a rare congenital cardiovascular condition in which some pulmonary veins, but not all, drain into the systemic circulation rather than into the left atrium (LA). This case report presents an extremely uncommon PAPVR variant with double drainage of the left upper pulmonary vein (LUPV) into the LA and superior vena cava (SVC) through a dilated vertical vein (VV) and innominate vein (IV) that leads to dilatation of the right ventricle and pulmonary hypertension. Surgical repair was the treatment of choice in this case.

**Learning Objectives**

• To understand the importance of extensive search for partial anomalous pulmonary venous return in patients with unexplained right ventricular enlargement encountered during echocardiographic evaluation.
• To emphasize the value of multimodal imaging, including MDCT and 3-dimensional reconstruction images, to confirm the diagnosis of PAPVR and to delineate the exact variant of pulmonary vein anomalies.
• To recognize the options of treatment in case of significant left-to-right shunt are either surgical correction primarily if associated with another congenital anomaly vs percutaneous catheter-based treatment in isolated PAPVR.

## History of Presentation

A 40-year-old man presented to the outpatient cardiology clinic in January 2024 with progressive episodes of shortness of breath and palpitation during the past 2 years. There was no chest pain and no history of syncope. There was no family history of cardiac disease or sudden cardiac death. He denied smoking and alcohol consumption and had a negative history of allergies.

## Past Medical History

The patient had no significant past medical history.

## Differential Diagnosis

**Figure 1:**
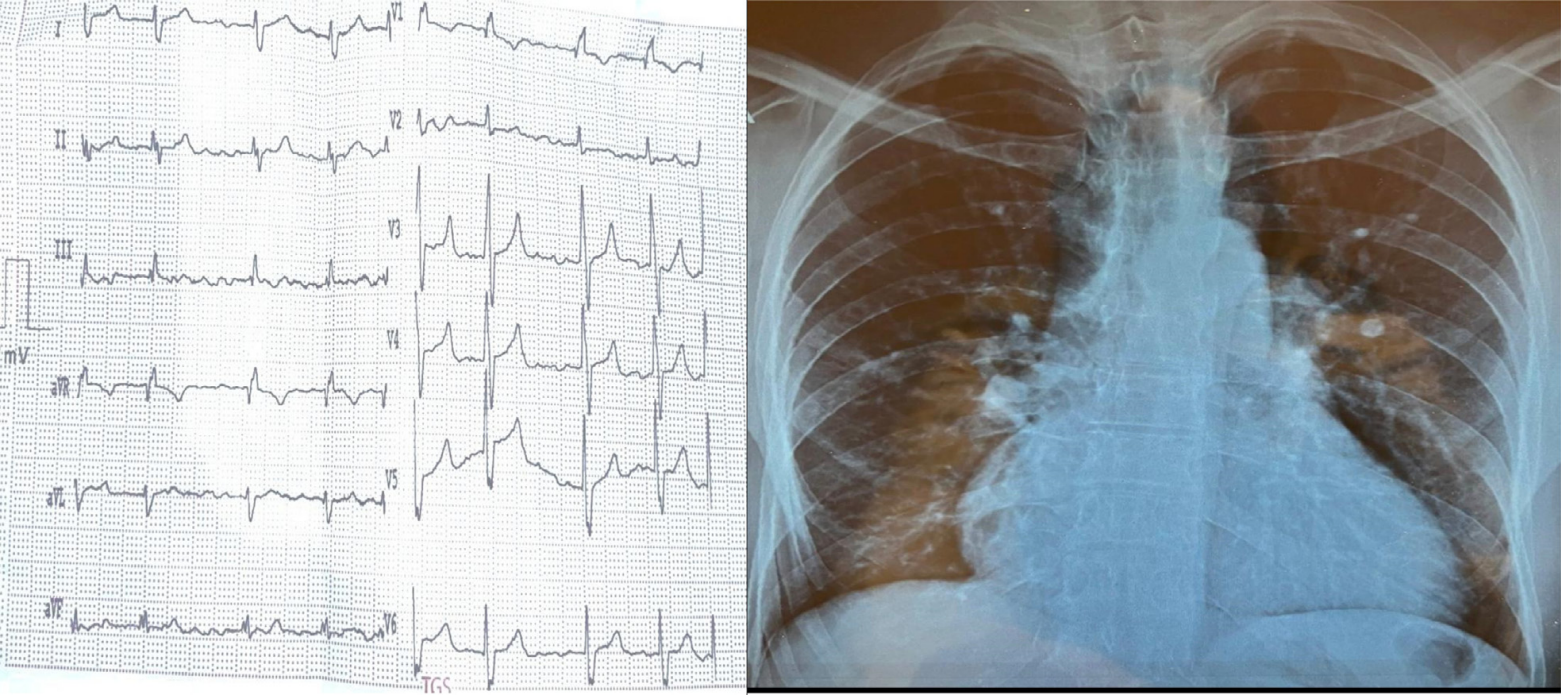
Electrocardiography Electrocardiography showed a pattern of right bundle branch block, whereas chest x-ray showed enlargement of the right cardiac border.

**Figure 2:**
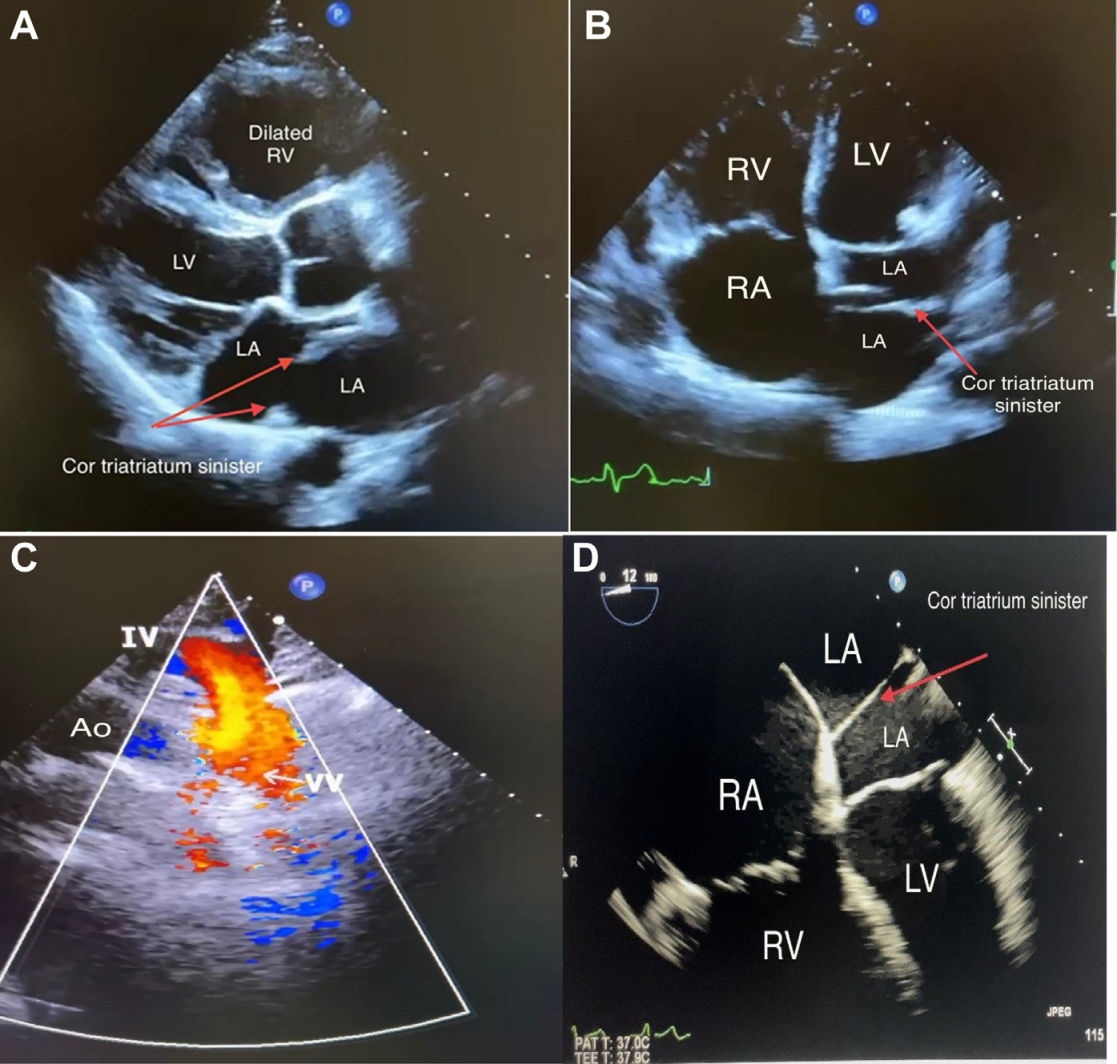
Transthoracic and Transesophageal Echocardiography (A, B, D) Significantly dilated RV and cor triatriatum sinister is shown: a thin fibromuscular membrane found inside the LA that causes division of the LA into 2 parts (red arrows). (C) VV: suprasternal view at the short axis of the aortic arch—a dilated vessel with a red color flow on color Doppler study suggestive of a venous structure with blood drainage into the IV. Ao = Aorta; IV = innominate vein; LA = left atrium; LV = left ventricle; RA = right atrium; RV = right ventricle; VV = vertical vein.

The patient’s vital parameters were stable, with blood pressure of 130/70 mm Hg, heart rate of 78 beats/min, and peripheral oxygen saturation at 95%. Cardiovascular and chest examinations were unremarkable. There was no lower limb swelling. His resting electrocardiogram showed a pattern of right bundle branch block, whereas chest x-ray revealed enlargement of the right cardiac border (Figure 1). Transthoracic echocardiography showed normal left ventricular size and systolic function and dilated right ventricle with an estimated systolic pulmonary pressure of 50 mm Hg. There was no evidence of atrial septal defect (ASD) or any other intracardiac shunt and no valvular abnormality. A linear tissue-like density subdividing the LA into proximal and distal chambers demonstrated a picture of cor triatriatum sinister (CTS) (Figures 2A, 2B, and 2D). Color flow Doppler showed the communication between left atrial subdivisions. The suprasternal view demonstrated a dilated vessel with a red color flow on color Doppler study, suggestive of a venous structure with blood flow drainage into the IV in favor of a VV (Figure 2C). Transesophageal echocardiography showed an unexpectedly normal LUPV connection to the LA with no evidence of ASD. The dilated right side of the heart with no evidence of intracardiac communication, in addition to the presence of a VV that drains into the IV, raised the suspicion of the presence of left-sided PAPVR, despite LUPV having a normal connection to the LA.

## Investigations

**Figure 3:**
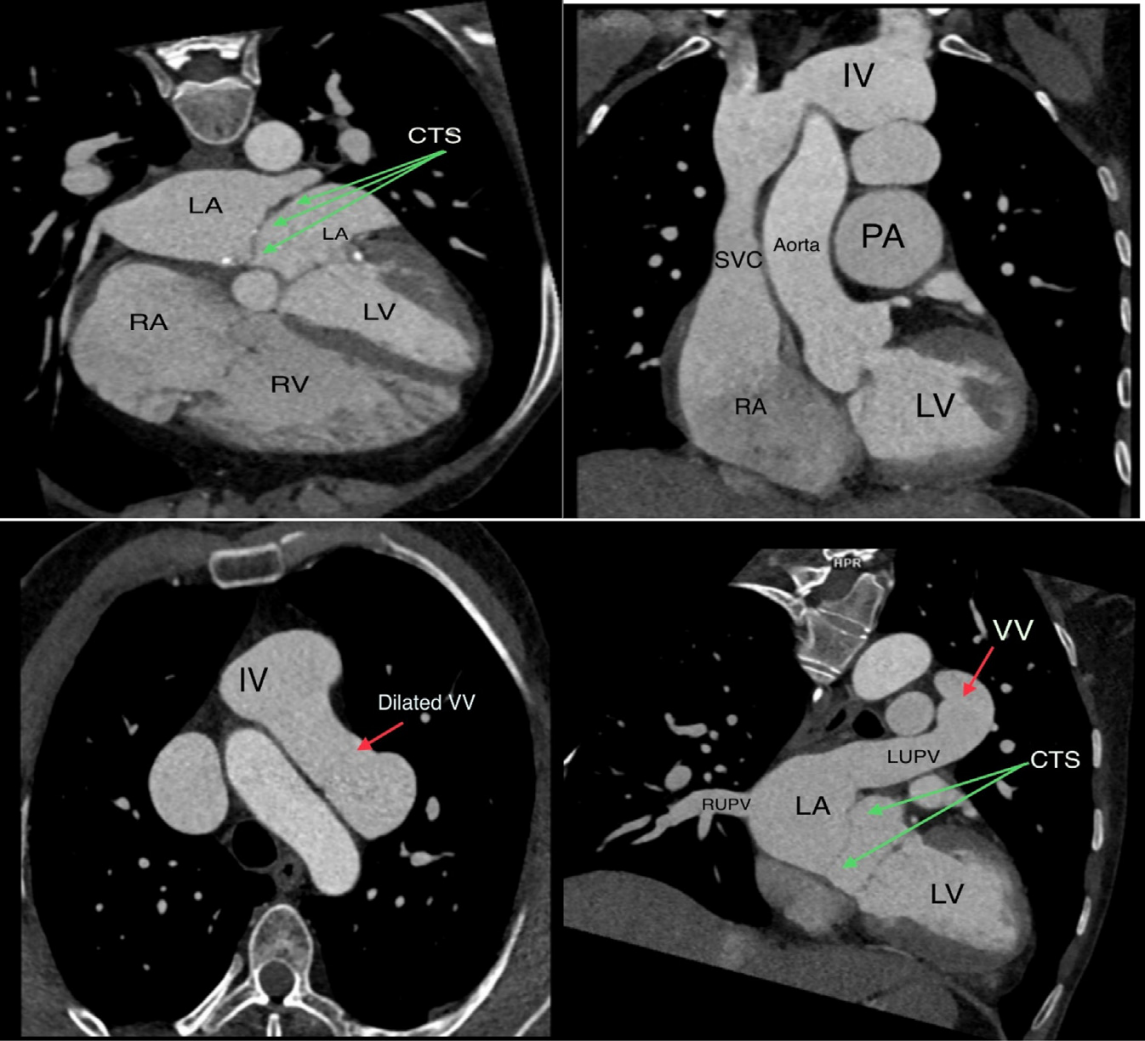
Contrast-Enhanced Multidetector Computed Tomography Contrast-enhanced multidetector computed tomography demonstrated dilated right side of the heart and dilated VV (red arrow) that terminated into the IV. Left atrium seen also divided into 2 cavities by CTS (green arrows). CTS = cor triatriatum sinister; LUPV = left upper pulmonary vein; RUPV = right upper pulmonary vein; SVC = superior vein cava; other abbreviations as in Figure 2.

**Figure 4:**
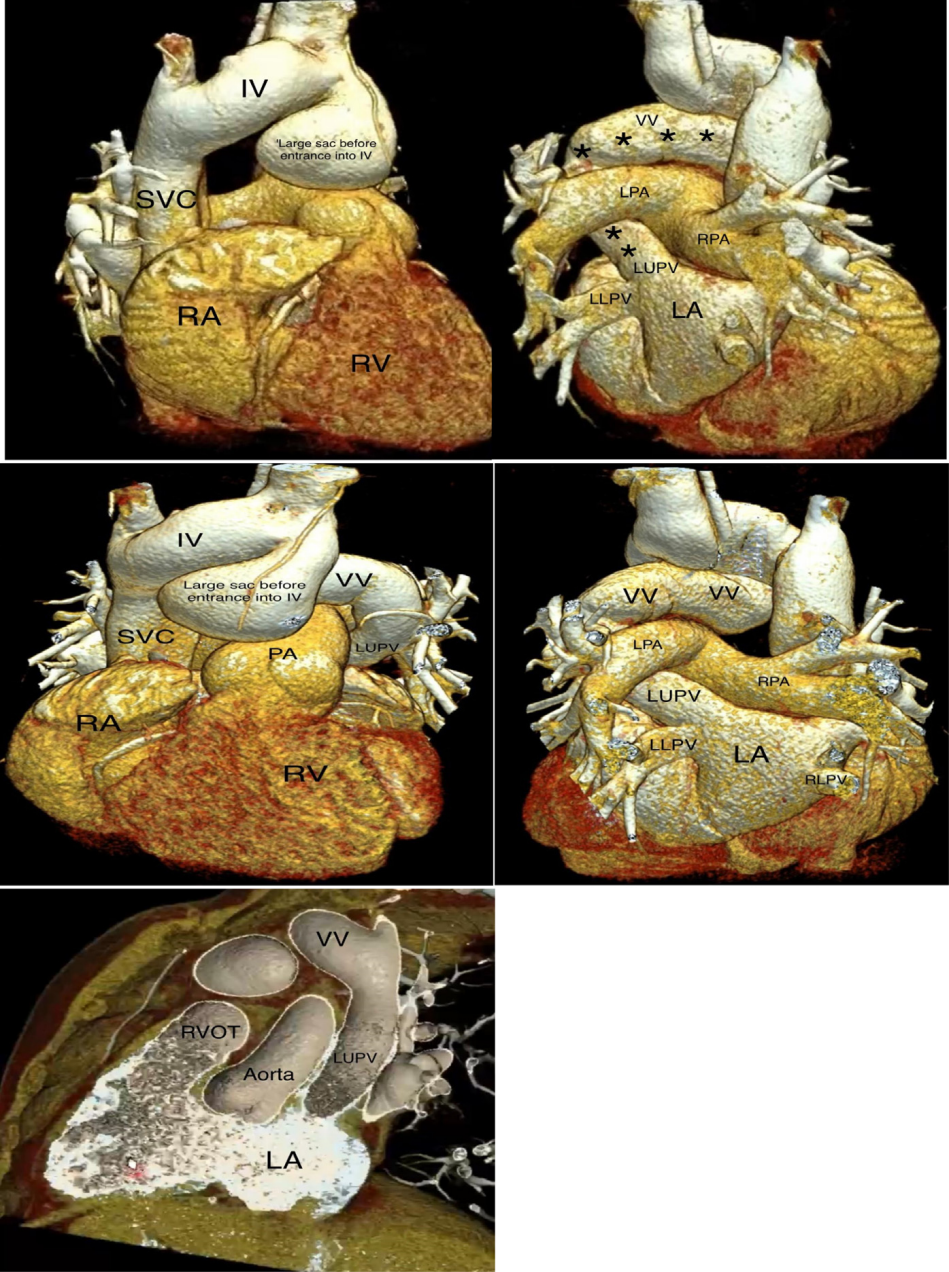
3-Dimensional Volume-Rendered Reconstruction Images Three-dimensional volume-rendered reconstruction images of cardiac computed tomography demonstrated the LUPV with a double drainage into the LA and dilated tortuous VV (solid stars show the track connection of LUPV and VV) that directed to a large sac before entrance into the IV and then the SVC. LLPV = left lower pulmonary vein; LP = left pulmonary artery; PA = pulmonary artery; RLPV = right lower pulmonary vein; RPA = right pulmonary artery; RVOT = right ventricular outflow tract; other abbreviations as in Figures 2 and 3.

A contrast-enhanced cardiac multidetector computed tomography (MDCT) showed 4 pulmonary veins with normal drainage into the LA; however, the LUPV had another drainage directed (double drainage) into a dilated tortuous VV that went into a large sac before entrance into the IV, which drains finally into the SVC and right atrium (RA) (Figure 3). Three-dimensional reconstruction images of cardiac computed tomography revealed the double connection of the LUPV with the LA and a dilated VV that directed to a large sac before entrance into the IV (Figure 4). The other pulmonary veins had normal drainage into the LA.

## Management

**Figure 5:**
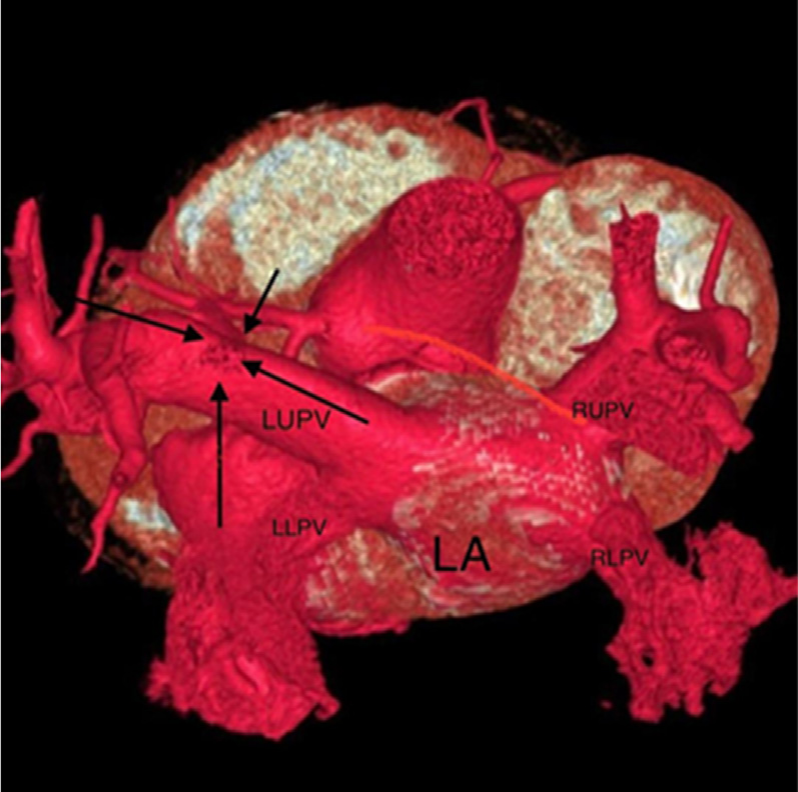
3-Dimensional Volume-Rendered Reconstruction Computed Tomography Three-dimensional volume-rendered reconstruction computed tomography postoperative correction showed single drainage of the LUPV into the LA and a repaired site of the LUPV seen (solid arrows) without vertical vein. RUPV = right upper pulmonary vein; other abbreviations as in Figure 2, Figure 3, Figure 4.

Because the right ventricle had become enlarged and the patient was symptomatic, the patient underwent a surgical repair consistent with resection of the obstructive membrane of the CTS in addition to resection of a dilated VV and repair of the LUPV. Cardiac computed tomography postoperative correction showed single drainage of the LUPV into the LA (Figure 5). The patient was discharged on the fourth day postoperatively in good condition.

## Discussion

The abnormal connection of 1 to 3 pulmonary veins to the RA or its tributaries is a rare congenital cardiovascular anomaly known as PAPVR.^1^ The lack of normal connection of some pulmonary veins with the LA and rather connection with other systemic veins, including the SVC, inferior vena cava, and/or direct connection with the RA, is considered the primary pathologic abnormality in common PAPVR types with a prevalence of 0.4% to 0.7%.^2^ The normal pulmonary vein development that occurs early in embryonic life is a complicated process, and a failure at any level of this process may lead to different types of anomalies in the connection of pulmonary veins.^3^ All types of PAPVR result in left-to-right shunt, volume overload, and enlargement of the right-sided cardiac chambers. Left-sided PAPVR usually drains through a VV and IV to the SVC and RA,^4^ whereas right-sided anomalous pulmonary veins usually have abnormal connections to the SVC, RA, and coronary sinus or drain to the inferior vena cava via a scimitar vein. The clinical presentation varies widely from asymptomatic patients to congestive heart failure. Up to 75% of PAPVR is associated with other congenital heart disease. A prevalent association is with sinus venosus ASD. Cor triatriatum has an association of 10% to 33% with PAPVR.^5^ Echocardiography is considered the initial imaging technique of choice.^6^ In some cases, by identifying the VV draining into the IV, echocardiography may directly reveal left-sided PAPVR. Contrast-enhanced MDCT is an excellent imaging modality in detecting vascular structures peripheral to the heart in the thorax,^7^ with a detection rate that approaches 100% using axial and 3-dimensional reconstructed images.^8^ Magnetic resonance imaging is also a preferred imaging modality for the assessment of the LA and pulmonary veins. It can detect pulmonary vein anomalies connection in addition to complex cardiovascular anomalies.^9^

A very rare type of PAPVR is when a portion of the lung through a pulmonary vein drains to both the LA and a systemic venous structure, giving rise to anomalous dual pulmonary venous drainage. Patients with anomalous dual drainage may be asymptomatic or may present later in life with congestive heart failure. In the literature, only very few cases have been reported. Surgical correction is usually the treatment of choice in case of a significant left-to-right shunt, especially if associated with another congenital anomaly, as in most patients. The very rare instances of isolated dual drainage of anomalous pulmonary veins may be a feasible option for catheter-based treatment through percutaneous occlusion of abnormal PAPVR flow using a coil or Amplatzer vascular plugs (Abbott Cardiovascular).^10^

Here, we report a symptomatic man with a combination of a very rare variant of dual drainage of left-sided PAPVR and CTS treated successfully through surgical correction for both anomalies that resulted in improvement of general condition and functional capacity.

## Follow-Up

At 1-month clinic visit, the patient stated that palpitations had disappeared and functional capacity had greatly improved. Follow-up transthoracic echocardiography showed much improvement in the size of the right ventricle and normal systolic pulmonary pressure with no evidence of VV on Doppler study.

## Conclusions

Dual drainage of PAPVR is a very rare variant of pulmonary veins anomalies. Unexplained right ventricular enlargement encountered during echocardiographic evaluation should always encourage an extensive search for PAPVR. Echocardiography, MDCT, and magnetic resonance imaging are most useful in the evaluation of patients to delineate the exact variant of pulmonary vein anomalies.

## Funding Support and Author Disclosures

The authors have reported that they have no relationships relevant to the contents of this paper to disclose.
